# In Vitro Study of Influence of Au Nanoparticles on HT29 and SPEV Cell Lines

**DOI:** 10.1186/s11671-017-2264-9

**Published:** 2017-08-15

**Authors:** Elena Pavlovich, Nataliia Volkova, Elena Yakymchuk, Olena Perepelitsyna, Michail Sydorenko, Anatoliy Goltsev

**Affiliations:** 1 Institute for Problems of Cryobiology and Cryomedicine, National Academy of Science of Ukraine, Pereyaslavskaya str., 23, Kharkiv, 61015 Ukraine; 2 Department for Biotechnical Problems of Diagnostic, Institute for Problems of Cryobiology and Cryomedicine, National Academy of Science of Ukraine, 42/1Nauky av., Kiev, 03028 Ukraine

**Keywords:** 61.46+w, 61.48+c, 61.48De, 87.15−v, 87.64−t

## Abstract

Cell culture models are excellent tools for potential toxicity of nanoparticles and fundamental investigations in cancer research. Thus, information about AuNP potential toxicity and effects on human health is necessary for the use of nanomaterials in clinical settings. The aim of our research is to examine the effects of AuNPs on the epithelial origin cell lines: continuous and oncogenic. Embryonic porcine kidney epithelial inoculated (SPEV) cell line and colorectal carcinoma cell line (HT29) were used. In the test cultures, the cell proliferation, necrosis/apoptosis, and multicellular spheroids generation were evaluated. We demonstrated that AuNP concentrations of 6–12 μg/ml reduced the proliferation of SPEV and HT29 cells and increased the cell number at early and late stages of apoptosis and necrosis. It was shown that small concentrations of AuNPs (1–3 μg/ml) stimulate multicellular spheroid formation by HT29 and SPEV cells. However, higher AuNP concentrations (6–12 μg/ml) had both cytotoxic and anti-cohesive effects on cell in suspension. The large sensitiveness to the action of AuNPs was shown by the line of HT29 (6 μg/ml) as compared to the SPEV cells (12 μg/ml). This experimental study of the effect of AuNPs on SPEV and HT29 cell lines will justify their further application in AuNP-mediated anticancer treatment.

## Background

Production and investigation of the gold nanoparticles (AuNPs) have not only high potential for wide therapeutic application of gold [1, 2] but also made them suitable for specific biomedical applications such as target therapies [3, 4]. Recent reports have demonstrated that the use of AuNPs provides an opportunity for novel antitumor therapies with a reduced risk for development of resistance. Thus, several studies have proven nanoparticle antitumor activity against breast, liver, gastric, colon, lung cancer [5, 6].

It is known that nanoparticles (NPs) can modulate cell fate, induce or prevent mutations, initiate cell–cell communication, and modulate cell structure [7, 8]. In addition, AuNPs have advantages over other metal NPs due to their biocompatibility and antitumor activity [8–12]. The cytotoxic and genotoxic effects of AuNPs are associated with their shape, size, charge, concentration, interaction time, etc. [12–14]. Thus, information about their potential toxicity and effects on human health is necessary for the use of nanomaterials in clinical settings.

Currently, despite the great success in the targeted therapy, the problem of selective delivery of AuNPs in the target tissue remains unsolved. Some studies have noted different rates of uptake of NPs by epithelial cells of different origin [15, 16]. Yet, investigations to explain this phenomenon are lacking even though they may help to achieve tissue-selective targeting of AuNPs. Anatomical or physiological differences between different epithelia could explain differences in AuNPs uptake and transport rates. In particular, the rate of uptake may be influenced by the plasma membrane properties of the cells and the binding of nanoparticles to cell surface glycoproteins and proteoglycans, as well as the cells’ capacity for vesicular transport [17]. Thus, taking into account the impossibility of exclusively selective interaction of nanoparticles with target cells, the comparative study of the features of their interaction with normal and oncogenic cells in order to avoid undesirable consequences of cancer therapy is topical [8–10].

Although in vivo models are valuable for evaluating biological toxicity of nanoparticles, cell culture models are highly useful for preclinical physiological and toxicological studies. Currently, cell cultures are widely used in various fields of biology, medicine, veterinary medicine, and biotechnology. The use of cell cultures allows to explore biological processes that are difficult and sometimes impossible, to study at the level of organisms. An important role of cell cultures is played in biotechnology in the production of many vaccines, test systems, and biologically active substances. Cell cultures are used to diagnose diseases of various etiologies, as test objects when testing new pharmacological, therapeutic, and cosmetic agents, as well as food additives [18].

In this work on cell culture models, we tried to examine the features of effects of AuNPs of the epithelial cells of continuous and oncogenic cell line origin. Monolayer culture of epithelial cells SPEV (embryonic porcine kidney epithelial inoculated line) and HT-29 (colon carcinoma cell line) cells can be considered as a model of normal and cancer epithelial tissues when anti-tumor therapy with AuNPs is applied. Several traditional cytotoxicity assays, including the adhesion, proliferation, necrosis/apoptosis, and multicellular spheroids were employed to validate the cell cytotoxicity of AuNPs.

## Methods

### Culture of SPEV Cells

SPEV cells were cultured in plastic flasks in DMEM (Sigma, USA) with 5% FCS (*v*/*v*) (HyClone, USA) supplemented with penicillin/streptomycin (PAA, Austria) and amphotericin B (5 μg/ml) (5% CO_2_, 95% humidity) as reported by [19]. Seeding concentration was 0.5–2 × 10^4^ cells/cm^2^. Culture medium was replaced every 2 days. Cells were passaged at 100% confluence [20]. SPEV cell line grew and preserved initial morphological structure of monolayer during serial passages without evidence of cell degeneration in culture.

### Culture of HT29 Cells

HT29 cells were cultured in plastic flasks (Nunc, Denmark) in RPMI-1640 medium (Sigma, USA) with 10% FCS (*v*/*v*) (HyClone, USA) supplemented with 2 mM L-glutamine (Sigma, USA) and 40 mg/ml gentamycin (Sigma, USA) under standard conditions (5% CO_2_, 95% humidity) [21]. The optimal cell density was 0.5–4.0 × 10^4^ cells /cm^2^. The cells were kindly provided to us by the Bank of Cell Lines from Human and Animal Tissue of RE Kavetsky Institute of Experimental Pathology, Oncology and Radiobiology NAS of Ukraine.

### Manipulations with AuNPs

**Fig. 1:**
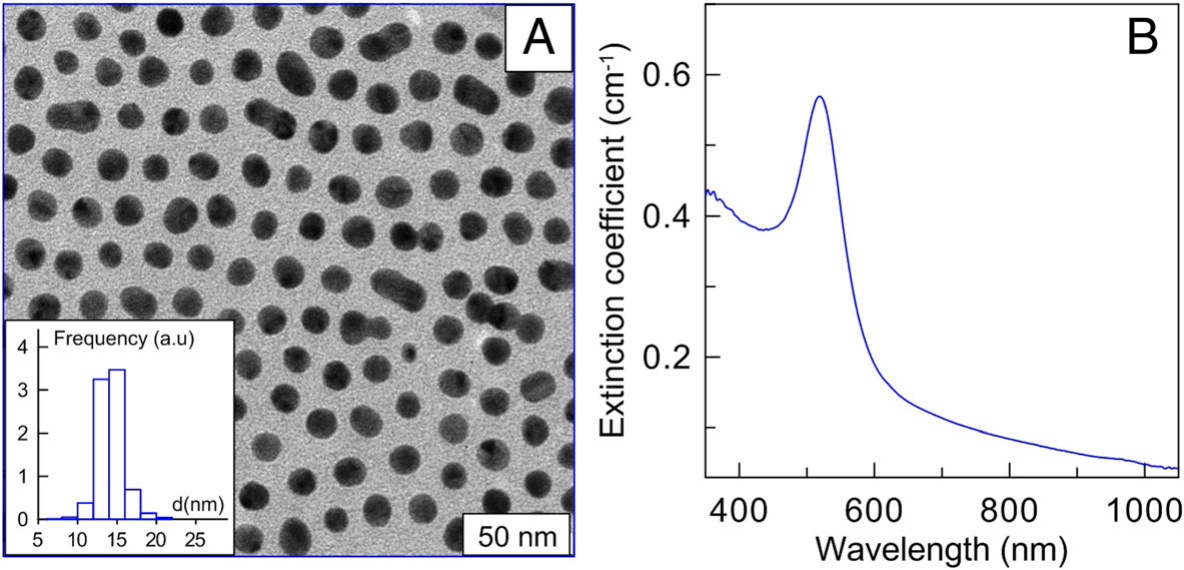
Results of dark field electron microscopy, **a** image of 15 nm AuNPs (note––diagram of the size distribution), **b** extinction spectra of 15 nm AuNPs

AuNPs were kindly provided by the Institute of Biochemistry and Physiology of Plants and Microorganisms, Russian Academy of Sciences. AuNPs were synthesized by citrate method [22]. The average size of nanoparticles was 15 nm. The initial concentration of gold was 57 μg/ml. The results of dark field electron microscopy, image of 15 nm AuNPs, and extinction spectra of 15 nm AuNPs (b) are shown in Fig. 1 (Fig. 1a; note––diagram of the size distribution) [23]. AuNPs were introduced in cells by passive diffusion at 37 °C. The investigated concentrations were 1, 3, 6, and 12 μg/ml. Cells without AuNPs under the same conditions were taken as control ones.

### Adhesion and Proliferation Cells

Morphofunctional state of cell cultures was judged by adhesive properties and proliferative activity. The adhesive properties of SPEV and HT29 cells were visually evaluated using an inverted microscope; the numbers of adhered and flattened cells were counted 30, 60, 120, 180, and 1440 min after culturing beginning.

The proliferation dynamics of SPEV and HT29 cells was studied for 1–4 days. To examine the increment of cell number in the studied cultures to the investigation terms, they were enzymatically (1:1 (0.25% trypsin solution: EDTA, PAA)) detached from plastic and the number of cells was counted. The total number of cultured cells was counted by the traditional method in Goryaev’s chamber.

### Apoptotic/Necrotic Processes

Apoptotic and necrotic processes in SPEV and HT29 cells exposed to AuNPs were investigated in 4 days with Annexin-V (BD, USA), 7-Amino-Actinomycin (7AAD) (BD) dyes using a FACS Calibur Becton-Dickinson. The results were analyzed with WinMDI v.2.8 software.

### Multicellular Spheroids

The multicellular spheroids (MSs) were generated to estimate the in vitro impact of AuNPs on migration and aggregation potential of investigated cells. Spheroid (3-D) model system of SPEV and HT29 cells was cultured by the conventional method, which was reported by [24] and modified in our laboratory [25]. Briefly, cell suspension were counted using trypan blue and equal numbers of cells (5 × 10^4^ cells/cm^2^) were planted in full supplemented culture medium. MS generation was achieved by handling cell culture with 0.24% carboxy-methyl-cellulose (CMC) in 24-well plates coated with 1% agar with rotation (80 rpm) for 24 h. After that, 3-D cell culture was maintained in standard conditions. To investigate dependence of the size and number of MSs on the AuNP concentration, MSs were generated in the presence of AuNPs. Further cultivation was conducted for 48 h with constant rotation of plates. At the next stage, micro photo images were made by dark field method with a Carl Zeiss Stemi 2000 microscope. MS morphology was studied with the help of Axio Vision Release 4.7 program (Zeiss). This program allows measuring the geometric dimensions of cell aggregates. Then, the volume of all MSs, which were in the files, was calculated. It was used with the following formula: *V* = 0.4 × *a* × *b* ^2^, where *a* and *b*––geometric diameters of MSs [24]. For statistical analysis, all cell aggregates were grouped by size from 1 × 10^−4^ mm^3^ to 1 × 10^−2^ mm^3^ with increment of 1 × 10^−3^ mm^3^. The MS number and median of MS volumes were estimated for each group.

## Statistical Analysis

A single-factor analysis of variance and Student’s *t* test were used for statistical processing of the data with the software package Statistica 8. The significance threshold was 0.05. The results are presented as means and standard errors (M ± SE).

## Results

### Effect of AuNPs on Adhesion of SPEV and HT-29 Cells

**Fig. 2:**
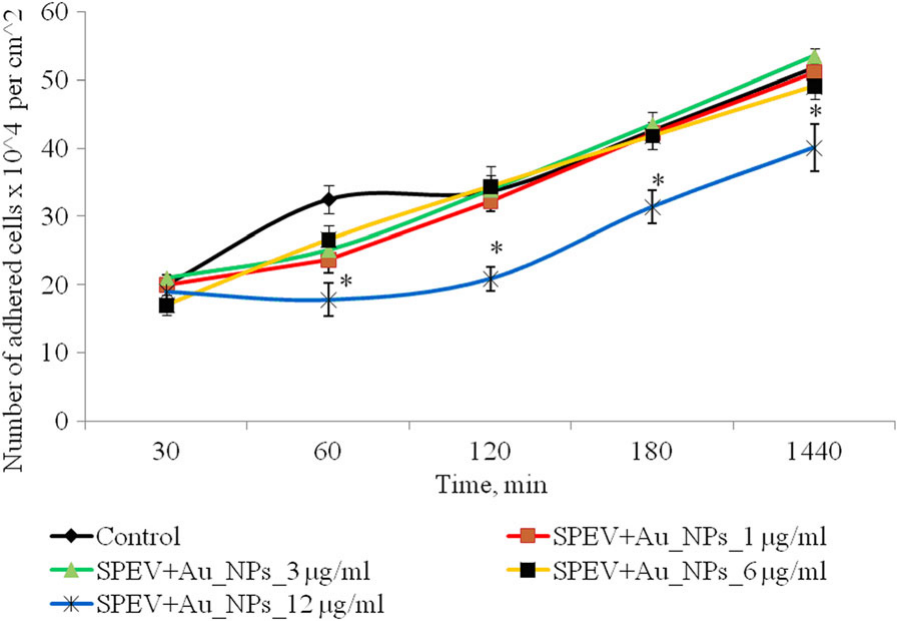
Dynamics of adhesion of SPEV cells after exposure of AuNPs, *p ≤ 0.05 is significant versus with the control

Cell adhesion is an indicator of functional state of cells, and it is necessary for further growth of culture. When adhesion terminated, cells became flattened and gained appropriate morphology. Adhesive properties of SPEV cells are presented in Fig. 2.

**Fig. 3:**
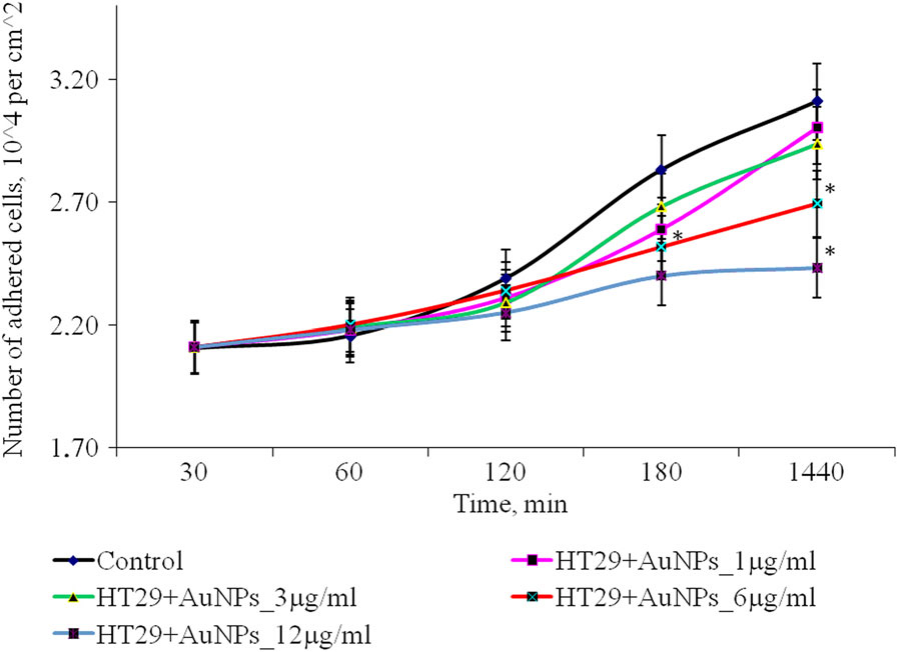
Dynamics of adhesion of HT29 cells after exposure of AuNPs, *p ≤ 0.05 is significant versus with the control

After 1 h cultivation of SPEV cells with AuNPs at 1, 3, and 6 μg/ml, the number of adhered cells was lower versus the control value. The percentage of flattened cells in samples with AuNPs for these concentrations did not significantly differ from the control. Adhesion was slowed down after 1 h incubation with AuNPs at 12 μg/ml. The number of adhered cells per squared centimeter was reduced by 1.8 times versus the control. This tendency in adhesion persisted for all the test periods. After 24 h of observation, the number of adhered cells was lower versus the control by 1.3 times. At the same time, incubation of AuNPs at small concentrations (1 and 3 μg/ml with tumor cells (HT29) had no significant effect on the amount of adhesive cells. Increasing of AuNP concentration to 6 and 12 μg/ml lead to decreasing the number of tumor cells in the adhesive fraction in 1.16 and 1.28 times, respectively, (Fig. 3). The obtained data can be influenced by several processes. The one is the cytostatic/cytotoxic effect of AuNPs on the adhesion fraction of both tumor and embryonic cell lines, which leads to cell death, transition to apoptosis, or necrosis. The other process is the reduction of cell adhesion, under the influence of AuNPs and transfer of cells into the suspension fraction. Notably, both processes can be realized simultaneously, and each one can contribute to the decrease in the number of living cells in the adhesion fraction.

### Effect of AuNPs on Proliferation of SPEV and HT-29 Cells

**Fig. 4:**
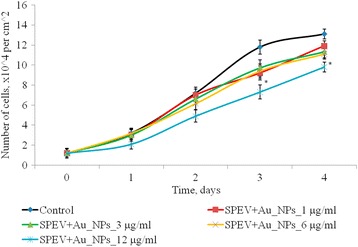
Proliferation of SPEV cells after exposure of AuNPs, *p ≤ 0.05 is significant versus with the control

The effect of AuNPs within the concentration range of 1–12 μg/ml on proliferative processes in SPEV cell culture was studied (Fig. 4). On days 2–4 of culturing with AuNPs at 1, 3, and 6 μg/ml, the cell number did not significantly differ from the control. On day 4 of culturing with AuNPs at 3 and 6 μg/ml, this index decreased by 1.15 and 1.23 times, respectively, as compared with the control. Reduction in the cell number by 1.5 times (days 2 and 3) and by 1.15 times on day 4 of culturing with AuNPs at 12 μg/ml was observed in SPEV culture versus the control. Thus, the AuNP concentration, 12 μg/ml, slowed down cell growth within the observed time period.

**Fig. 5:**
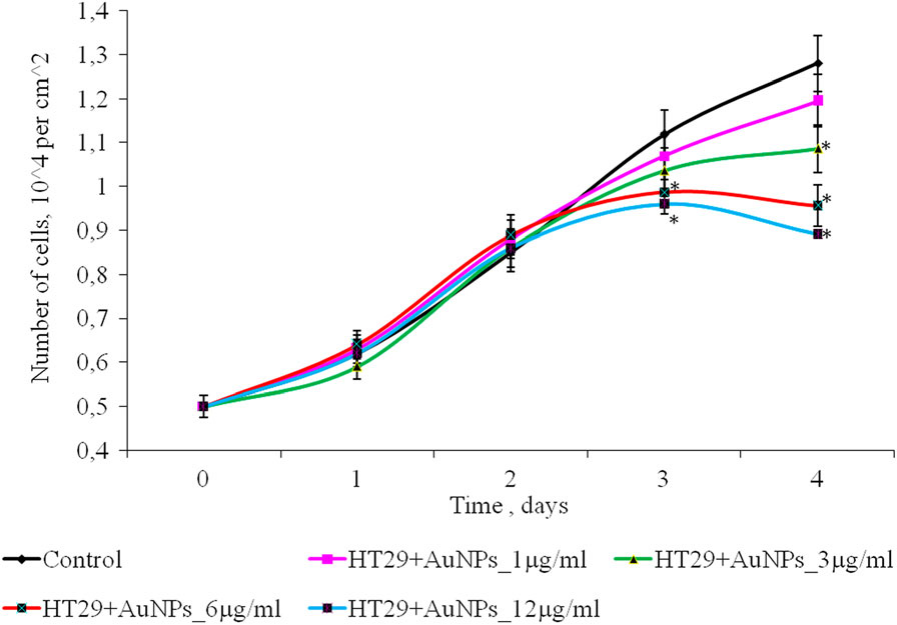
Proliferation of HT29 cells after exposure of AuNPs, *p ≤ 0.05 is significant versus with the control

The effect of AuNPs at concentrations from 1 to 12 μg/ml on the number of HT 29 cells in a monolayer culture is shown in Fig. 5. During the first 3 days of incubation, the number of cells in the control and in the presence of AuNPs was not statistically different. On the 4th day of cultivation, it was noted a dose-dependent decreasing of the number of cells in 2D culture. So, after 4 days of cultivation, for low concentrations of AuNPs (1 and 3 μg/ml), the number of HT 29 cells is not significantly different in comparison with control. But at higher AuNP concentrations (6–12 μg/ml), HT29 cell number was lower than the control in 1.33 and 1.44 times, respectively.

### Effect of AuNPs on Apoptotic/Necrotic Processes in SPEV and HT-29 Cells

SPEV and HT-29 cells in the presence of AuNPs were cultured for 4 days under the standard conditions. The culturing of SPEV and HT29 cells with AuNPs at 1 and 3 μg/ml and the indexes of apoptotic/necrotic processes did not significantly differ from the control (Tables 1 and 2).

**Table 1:** Cytofluorimetric analysis of SPEV cells after 4 days culturing with AuNPs, staining with Annexin V and 7AAD

Sample/region	Annexin *V* ^+^ /7AAD^−^	Annexin V^+^/7AAD^+^	Annexin *V* ^*−*^/7AAD^−^	Annexin *V* ^−^/7AAD^+^
Control	3.4 ± 0.4	4.5 ± 0.7	91.6 ± 1.0	0.5 ± 0.1
SPEV + Au_NPs_1 μg/ml	3.5 ± 0.6	4.4 ± 0.8	91.4 ± 1.2	0.7 ± 0.2
SPEV + Au_NPs_3 μg/ml	3.7 ± 0.5	3.6 ± 0.8	91.0 ± 1.1	1.7 ± 0.1
SPEV + Au_NPs_6 μg/ml	3.9 ± 0.6	10.1 ± 0.5*	84.6 ± 1.2*	1.4 ± 0.2*
SPEV + Au_NPs_12 μg/ml	5.1 ± 0.8*	12.5 ± 0.7*	81.1 ± 1.2*	1.3 ± 0.1*

Note*p ≤ 0.05 is significant versus with the control

**Table 2:** Cytofluorimetric analysis of HT29 cells after 4 days culturing with AuNPs, staining with Annexin V and 7AAD

Sample/region	Annexin V^+^ /7AAD^−^	Annexin V^+^/7AAD^+^	Annexin V^−^/7AAD^−^	Annexin V^−^/7AAD^+^
Control	3.1 ± 0.5	4.6 ± 0.6	91.8 ± 0.9	0.5 ± 0.2
HT29 + Au_NPs_1 μg/ml	2.9 ± 0.7	3.8 ± 0.5	92.8 ± 1.1	0.5 ± 0.3
HT29 + Au_NPs_3 μg/ml	3.0 ± 0.6	3.9 ± 0.7	92.5 ± 1.2	0.6 ± 0.2
HT29 + Au_NPs_6 μg/ml	4.1 ± 0.5*	7.7 ± 0.4*	87.3 ± 1.5*	0.9 ± 0.3*
HT29 + Au_NPs_12 μg/ml	5.3 ± 0.9*	9.2 ± 0.8*	84.0 ± 1.3*	1.2 ± 0.2*

Note *p ≤ 0.05 is significant versus with the control

Culturing with AuNPs at 6–12 μg/ml increased the percentage of Annexin V+/7AAD+, Annexin V−/7AAD+, and Annexin V+/7AAD-cells as well as reduced the percentage of alive cells. The number of Annexin V^+^/7AAD^+^ SPEV cells was higher than the control value by 7.8 ± 0.7% (*p* ≤ 0.05) with 12 μg/ml of AuNPs. The number of Annexin V^+^/7AAD^+^ HT 29 cells was higher than the control value by 3.2 ± 0.4% (*p* ≤ 0.05) with 6 μg/ml of AuNPs and by 4.8 ± 0.6% (*p* ≤ 0.05) with 12 μg/ml of AuNPs.

### Effect of AuNPs on Generation of Multicellular Spheroids from SPEV and HT29 Cells

**Fig. 6:**
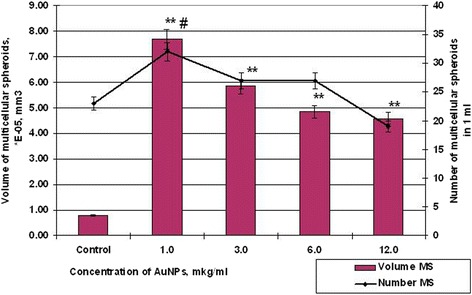
Number and volume of MS cells SPEV after incubation with AuNPs, #*p* ≤ 0.01 (for number MSs); **p ≤ 0.01 (for volume of MSs)

**Fig. 7:**
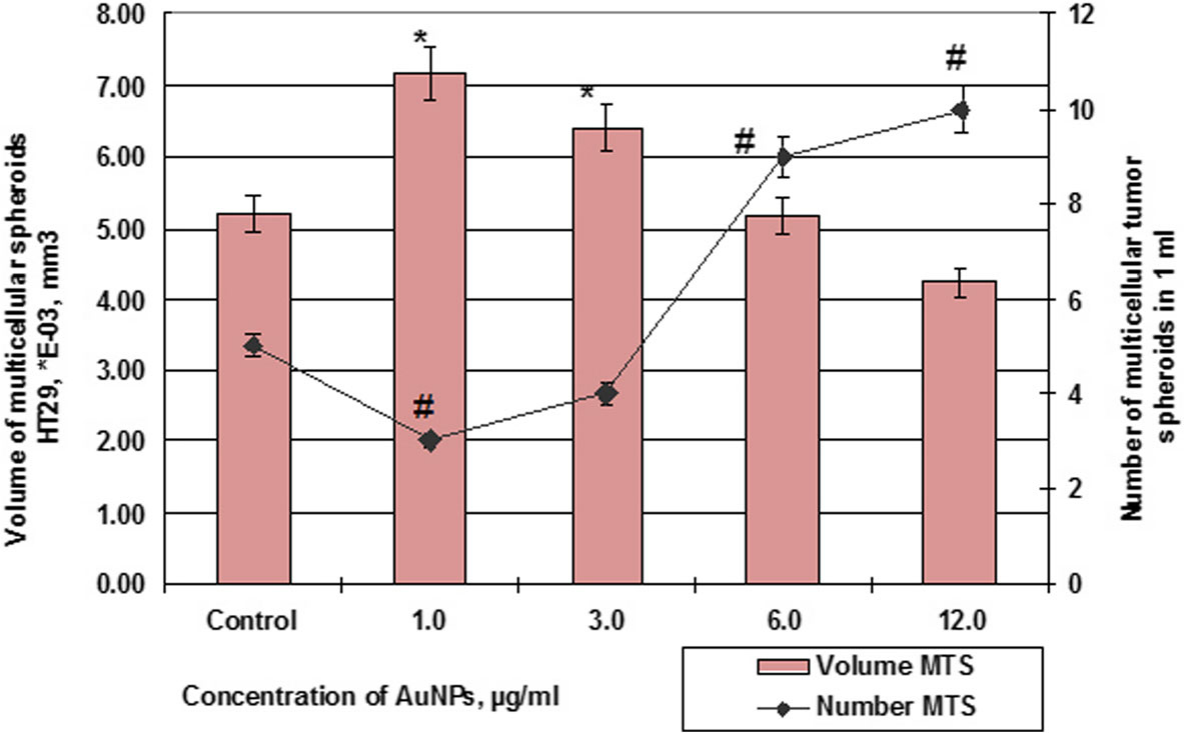
Number and volume of MS cells HT29 after incubation with AuNPs. #*p* ≤ 0.01 (for number MSs); **p ≤ 0.01 (for volume of MSs)

To determine the dependence of size and number of multicellular spheroids (MSs) on AuNP concentration, MSs were generated at various concentrations of AuNPs during 48 h. Our data demonstrated the variety ability of HT29 and SPEV cells to form multicellular spheroids under the same conditions of the microenvironment (Figs. 6 and 7).

So, if the control samples of HT29 cells for 48 h formed spheroids in average volume 5.19 × 10^−3^ mm^3^, the average volume spheroid of SPEV cells was 0.79 × 10^−5^ mm^3^. At the same time, the influence of AuNPs on the HT29 and SPEV cells had the same trend. The presence of AuNPs in the cell microenvironment stimulated the formation of multicellular spheroids in both cultures. Thus, when the concentration of AuNPs was 1 and 3 μg/ml volume of MSs for SPEV increased by 9.7 and 7.4 times, respectively, compared with the control (Fig. 6), the same AuNP concentrations also stimulated an increasing volume of MSs for HT29 by 1.4 and 1.2 times, respectively (Fig. 7).

Further increasing of AuNP concentration leaded to decreasing average volume of MSs in both cultures. The elevation in AuNP concentration from 1 to 12 μg/ml decreased the volume of HT29 MSs from 7.18 × 10^−3^ mm^3^ to 4.24 × 10^−3^, in 1.69 times, according to the control. As for SPEV, when the concentration of AuNPs was increased from 1 to 12 μg/ml, the volume of MSs decreased from 7.69 to 4.58 × 10^−5^ mm^3^, in 1.68 times, according to control. However, increasing AuNP concentration coincidences with reduction in volume of MSs and correlated with increases in the number of spheroids in culture of HT29 cells (Figs. 6 and 7). The number of HT29 MSs increased from 3 to 10 per field of view at AuNP concentration from 1 to 12 μg/ml. At the same time, the number of SPEV MSs decreased from 32 to 19, respectively.

The obtained data (Figs. 6 and 7) demonstrate that AuNPs are capable of influencing cohesive interactions in the cell-to-cell system. Our data show that small concentrations of AuNPs (1–3 μg/ml) stimulated the formation of multicellular spheroids of both embryonic and tumor cells. However, higher AuNP concentrations (6–12 μg/ml) had both cytotoxic and anti-cohesive effects on cell in suspension. This process contributed to formation of a larger number of HT29 MSs with the decreased average volume. As for SPEV, the high concentration of AuNPs may have a cytostatic effect which reduced cell numbers in the adhesive fraction and the number of MSs in suspension. Previously, the authors reported that carbon nanoparticles reduce the adhesion of cells to the substrate, stimulate cell transfer into the suspension, and leaded to formation of multicellular spheroids [25, 26]. In the literature, there are data about AuNP ability to break the structure of actin/myosin microfilaments and decrease cell proliferation, adhesion, and differentiation [27]. Our data confirmed this assumption.

## Discussion

We evaluated the effects of AuNPs on proliferation, necrosis/apoptosis, and formation of multicellular spheroids of the epithelial cells continuous and oncogenic cell line origin. It was shown that the AuNPs at 6–12 μg/ml reduced the number of SPEV and HT29 cells and increased the cell number at early and late stages of apoptosis and necrosis. The small concentrations of AuNPs stimulate formation of multicellular spheroids by HT29 and SPEV cells. However, higher AuNP concentrations had both cytotoxic and anti-cohesive effects on cell in suspension. The large sensitiveness to the action of AuNPs was shown by the line of HT29 (6 μg/ml) as compared to the SPEV cells (12 μg/ml.)

The effects of AuNPs on cellular morphology and cytoskeleton have only recently received more attention, and the underlying mechanism and forthcoming consequences have not been investigated in depth [28–30]. In this regard, it is important for all novel AuNP types to evaluate their endocytic uptake pathway and intracellular localization as a function of time. For different types of AuNPs, the effects have been described to be dependent on intracellular AuNP concentration and to be transient, where after recurrent cell divisions, the intracellular AuNP concentrations decrease exponentially and the effects are no longer observed. Also, possible endosomal escape of the AuNPs must be assessed. As cytoskeleton defects have been described to be clearly dependent on AuNP concentrations, a wide concentration range of particles should be tested in order to try and assess the maximal cellular loading capacity without any effects. Furthermore, as the cytoskeleton is also involved in many intracellular signaling pathways, it remains to be investigated whether the AuNPs induced cytoskeletal disruption leads to secondary effects [31].

As NPs have certain physical dimensions, the intracellular volume they occupy can lead to alterations in cellular morphology or affect the structure of the cellular cytoskeleton network [28, 29, 31]. The later effects can also be due to the high demands of the NP pose on the cellular endocytic manner. AuNPs have been described to have a profound effect on the morphology of several cell types, such as A549 human carcinoma lung cells [32]. AuNPs have also been described to have a concentration-dependent effect on the actin fibrils of human dermal fibroblasts [33, 34]. Mironava et al. [35, 36] further showed the cytoskeleton filaments to be disrupted as a function of AuNP exposure time, concentration, and size of the NPs although actin or β-tubulin protein expression levels were not affected.

The cell type used is also of great importance as different cell types, even when closely related, can react quite differently for the same type of nanomaterials [37, 38]. Preferably, those cell types which are most involved in the (future) biomedical applications of the NPs should be tested (e.g., epithelial, endothelial cells), or multiple cells which are derived from the different germ layers. When investigating cytotoxic effects, the use of cancer cell types should be minimized, as these can lead to aberrant results [39]. Cancer cells have several specific characteristics and altered intracellular signaling pathways which are destined to upregulate proliferation and maintain cell viability, which will make them less prone to some NP-mediated cytotoxic effects.

In our opinion, binding of AuNPs to surface functional groups (e.g., transmembrane proteins) of cells can be reversible or irreversible, resulting in temporary or permanent structural injuries [40, 41]. Potential implications of changes in biomechanical properties (e.g., hardness and elasticity), adhesiveness, and surface electrical properties of cells are perceivable. Thus, changes in hardness or elasticity are likely to influence the surface structural flexibility, production of mechanical energy for cell division, and cell motility. As for adhesiveness, the cell microenvironment is normally composed of extracellular matrix with specific molecules that allow cells to adhere to their surroundings [42]. Surface charge undoubtedly plays an important role in interactions between cells and their surroundings.

The other authors also reported of the NPs are preferentially localized in mitochondria and cause oxidative stress as well as potentiate structural damage [40]. A recent article by Pan et al. describes that 1.4 nm AuNPs induce necrosis via oxidative stress and mitochondrial damage in Hela cells [43]. Accumulation of nanoparticles in cell medium upon biodegradation is unsafe because it may disrupt organelles and even cause genetic mutations.

Changes occurring in cells during apoptosis are similar for most of cell types. In apoptotic cells, there are changes of lipid composition of plasma membrane: phosphatidyl serine transfers from cytoplasmic part of bilayer to outer side, causing caspase cascade activation, chromatin condensation, and disorder of electron transport chain in mitochondria and eventually arresting ATP synthesis. Programmed cell death can be triggered by receptor-mediated physiological stimuli resulted from genetic disorders, exposure to chemical or physical factors as well as by other changes in cells. We observed this effect is with 6–12 μg/ml of AuNPs.

Multicellular aggregates (spheroids, embryoid body) represent an intermittent level between monolayer growing cells and tissue culture. Spheroids are objective model of the cell three-dimensional growth and organization, the cell-to-cell interactions and influence of microenvironmental conditions, for example, AuNP concentration, on intensiveness of proliferation as well as on cell adhesiveness and formation of microaggregates. MS formation is a well-established culture method as for tumor as for embryonic cell lines [24, 44, 45]. In our work, formation and growth of spheroids is achieved by adding CMC as part of artificial extracellular matrix and surface coating by 1% agar which inhibited cell adhesion to surface and stimulated cell aggregation. At these conditions, MS culture can be realized until the aggregates forming central necrosis, due to limited cell mass growth or spontaneous differentiation of embryonic cells.

In literature, there is information about interaction AuNPs with colon cancer cell line and embryonic cell lines [46, 47]. According these data, exposure to even very low concentrations of AuNPs may have a damaging effect on the Human Embryonic Neural Precursor Cells and HT29 by stressing cell proliferation, differentiation, and apoptotic cell death.

There are published data that the effect of AuNPs is based on G0/G1 phase accumulation, S and G2/M phase depletion, as well as on reduced ATP levels in human oral squamous carcinoma cells (HSC-3) [48]. Cell cycle regulation may be addressed by violation of focal contacts of cells with substrate and cell transfer to suspended fraction in 2D culture and inhibition cell-to-cell contacts in gap junction in 3D culture [48–50]. Due to nanosize of AuNPs (near 15 nm), it cannot be centers of cohesion for cells. At the same time, intercalation of AuNPs into cell membrane [51], influence on cell membrane AuNP zeta potential [32], and influence on formation cell-to-cell/cell-to-surface contacts obviously can trigger mechanism for necrosis/apoptosis, cytotoxic effect, and cell cycle arrest. Violation of focal contacts of cells with substrate and cell transfer to suspended fraction is a way of cell cycle regulation [48, 49]. Small concentrations of AuNPs exerted no statistically significant cytotoxic effect on cells. However, higher AuNP concentrations had both cytotoxic and anti-cohesive effects on cell in suspension. This process contributed to formation of a larger number of MSs with the decreased average volume. We suppose that AuNP wedge into cohesive contacts of cells and compromise them. Thus, our experiments on the effects of AuNPs on SPEV and HT29 cell lines support their further application in development of AuNP-mediated cancer therapies.

Although future studies will be necessary to confirm anti-cancer effects on the in vivo animal studies. Nevertheless, our deep conviction is that if we know the nature of substance and its possible negative influence, we are able to avoid the detrimental effects of AuNP and use their positive biotechnological potential. Our investigation could be applied quite reliably in effective materials for anti-cancer treatment context with maximum advantage for medicine.

## Conclusions

Our results support the notion that AuNPs induce dose-dependent cytotoxicity in SPEV and HT29 cells. Furthermore, this report for the first time demonstrates that 15 nm AuNPs in concentrations of 6–12 μg/ml reduced the proliferation of SPEV and HT29 cells and increased the cell number at early and late stages of apoptosis and necrosis. Also, it was shown that small concentrations of AuNPs (1–3 μg/ml) stimulate formation of multicellular spheroids. However, higher AuNP concentrations had both cytotoxic and anti-cohesive effects on cell in suspension. The large sensitiveness to the action of AuNPs was shown by the line of HT29 (6 μg/ml) as compared to the SPEV cells (12 μg/ml.)
